# Investigation of the association of long-term NSAID use with radiographic hip osteoarthritis over four to five years: Data from the OAI and CHECK studies

**DOI:** 10.1016/j.ocarto.2023.100427

**Published:** 2023-12-09

**Authors:** Zubeyir Salis

**Affiliations:** a Division of Rheumatology, Geneva University Hospitals and Faculty of Medicine, University of Geneva, Geneva, Switzerland; b The University of Western Australia, School of Human Sciences, Perth, WA, Australia; c The University of New South Wales, Centre for Big Data Research in Health, Kensington, NSW, Australia

**Keywords:** Osteoarthritis, Hip, Non-steroidal anti-inflammatory drugs, NSAIDs, Radiographic hip osteoarthritis

## Abstract

**Objective:**

To examine the relationship between long-term use of non-steroidal anti-inflammatory drugs (NSAIDs) and the incidence and progression of radiographic hip osteoarthritis (RHOA), as well as the degeneration of individual radiographic features.

**Methods:**

We analyzed data from the Osteoarthritis Initiative (OAI) and the Cohort Hip and Cohort Knee (CHECK) study. Our exposure was the number of years of NSAID use over a 4-to-5-year follow-up period. Our outcomes were the incidence and progression of RHOA over a 4-to-5-year follow-up as assessed using a modified Croft grade in OAI and the Kellgren-Lawrence (K/L) grade in CHECK. The incidence of RHOA was defined as having RHOA (grade ≥2) at follow-up and investigated in “incidence cohorts” of hips without RHOA at baseline (grade <2). The progression of RHOA was defined as an increase of ≥1 grade at follow-up from baseline and investigated in “progression cohorts” of hips with RHOA at baseline (grade ≥2). Additionally, we assessed the degeneration of nine specific radiographic features, such as joint space narrowing and osteophytes, defined by a grade increase of ≥1 ​at follow-up from baseline, in all cohorts.

**Results:**

In the incidence cohorts, there were 5153 hips in OAI and 1011 in CHECK; in the progression cohorts, there were 285 and 106 hips, respectively. There was no association between NSAID use and the outcomes investigated.

**Conclusion:**

Over 4-to-5 years, long-term NSAID use showed no association with the incidence or progression of RHOA, or with the degeneration of individual radiographic features.

## 1 Introduction

Hip osteoarthritis (OA) is a chronic condition characterized by progressive loss of articular cartilage, osteophyte formation, subchondral cysts, and muscle weakness. These changes result in pain, stiffness, and reduced mobility [1]. It is a prevalent condition in the aging population. In the United States of America (USA) alone, it is estimated that 26.6 ​% of adults aged ≥45 years have radiographic hip OA, with 9.2 ​% experiencing symptomatic hip OA [2]. Globally, the prevalence of hip OA has increased significantly, showing a 115.40 ​% rise (from 0.74 million to 1.58 million) between 1990 and 2019 [3]. Despite its prevalence, there are no approved medications that can slow, delay, or reverse the structural changes in the hip joint due to OA [4].

Symptom management is crucial in hip OA treatment, with non-steroidal anti-inflammatory drugs (NSAIDs) being the most commonly used pharmacological treatment for pain relief [5]. However, NSAIDs are associated with significant risks, including gastrointestinal complications [6], increased risk of cardiovascular disease [7], and renal failure [8]. Consequently, international guidelines recommend their short-term use [[9], [10], [11], [12]]. Nonetheless, NSAIDs are often used long-term; a recent study indicated that the average duration of NSAID treatment among people with OA is about 16 months over three years [13].

There is concern that NSAIDs while alleviating symptoms of hip OA, might paradoxically contribute to the progression of this condition. By masking pain, NSAIDs may lead individuals to inadvertently exert more stress on their hip joints, potentially exacerbating the incidence and progression of hip OA. The exact impact of NSAID use on structural changes in hip OA remains unclear, with current literature providing mixed findings [[14], [15], [16], [17]]. Our study aims to investigate the association between long-term NSAID use and structural changes in hip OA over a follow-up period of 4–5 years. We utilized data from two prospective cohort studies, the Osteoarthritis Initiative (OAI) [18] from the USA and the Cohort Hip and Cohort Knee (CHECK) study [19] from the Netherlands, for this investigation.

We hypothesized that long-term NSAID use may negatively affect the structure of the hip joint. If confirmed, this would suggest that NSAID use, in addition to the natural progression of OA, could pose an additional risk of structural damage to the hip joint. The findings from this study will provide insights into the long-term effects of NSAID use on hip OA and will be instrumental in informing clinical practice.

## 2 Method

### 2.1 Data sources and ethics

We utilized data from the publicly available databases of two cohort studies: the Osteoarthritis Initiative (OAI) and the Cohort Hip and Cohort Knee (CHECK). Both cohorts predominantly included participants with or at risk of knee OA, with the CHECK cohort also including those at risk of hip OA. Ethical approval and informed consent were obtained as part of the original studies.

### 2.2 Exposure

The exposure in this study was defined as the number of years of NSAID use. In the OAI cohort, NSAID use was determined using the medication inventory method [20], where participants bring their current medications to a study visit for documentation. In the OAI cohort, NSAID use, including COX2 inhibitors, was identified if participants reported oral or capsule use within the last 30 days at each assessment. In the CHECK cohort, NSAID use, including COX2 inhibitors, was assessed during clinic interviews, with participants being classified as users if they confirmed using specific NSAIDs (ibuprofen, diclofenac, naproxen, celecoxib, rofecoxib) for hip and/or knee complaints at the time of assessment.

The duration of NSAID use was evaluated based on consecutive annual follow-ups. For instance, 1-year use was defined if NSAID use was reported at two consecutive annual follow-ups. The OAI cohort had annual follow-ups from year 1–4, while the CHECK cohort had follow-ups from year 1–5. Participants were classified as NSAID non-users if they reported no NSAID use at all visits, including baseline and subsequent follow-ups.

### 2.3 Outcomes

Primary outcomes included the incidence and progression of radiographic hip OA, utilizing radiographic data from baseline and the final follow-up (year 4 in the OAI, year 5 in the CHECK study). The OAI had hip radiographic data available only at four years, and the CHECK study had hip radiographic data at five years. Secondary outcomes were the incidence of end-stage hip OA, and the incidence and resolution of symptomatic hip OA.

Radiographic hip OA was assessed using two approaches. First, overall radiographic hip OA, scored using a modified Croft grade [21] in the OAI and the Kellgren-Lawrence (K/L) grade [22,23] in CHECK, was examined. Both grades use a grading system of 0–4, where higher numbers indicate worsened radiographic hip OA. We hereafter refer to both grades as ‘overall grade’. We considered a hip with an overall grade of ≥2 as having ‘radiographic hip OA’, and a hip with an overall grade of <2 as being ‘without radiographic hip OA’.

Second, we investigated nine individual radiographic features of hip OA. The grades for these nine individual radiographic features of hip OA ranged from 0 to 3, with higher numbers indicating worsened osteoarthritis of that individual radiographic feature. We hereafter refer to these as ‘individual grades’. These nine individual radiographic features were joint space narrowing (JSN) in 2 locations (lateral and medial); osteophytes in four locations (acetabular superior; acetabular inferior; femoral superior; and femoral inferior); cysts in one location (acetabular subchondral); sclerosis in one location (femoral subchondral); and deformity in one location (femoral head). Additionally, we investigated the sum of the individual grades for osteophytes in all four locations. This additional investigation aimed to increase the power to detect any association between long-term use of NSAIDs and osteophytes because of the difficulty in detecting small osteophytes in some locations radiographically [24]).

We defined the incidence of radiographic hip OA as moving from an overall grade of <2 ​at baseline to an overall grade of ≥2 ​at 4-to-5-year follow-up. In order to investigate the incidence of radiographic hip OA, we created an OAI incidence cohort and a CHECK incidence cohort that consisted of hips without radiographic hip OA at baseline. We defined the progression of radiographic hip OA as an increase of one or more overall grades at the 4-to-5-year follow-up from baseline. In order to investigate the progression of radiographic hip OA, we created an OAI progression cohort and a CHECK progression cohort that consisted of hips with radiographic hip OA at baseline.

We defined the degeneration of individual radiographic features as an increase of one or more individual grades at the 4-to-5-year follow-up from baseline. For the sum of the individual grades for osteophytes in all four locations, we defined the degeneration of individual radiographic features as an increase of two or more in the sum of the individual grades for osteophytes in all four locations from baseline at the 4-to-5-year follow-up [25]. The degeneration of individual radiographic features was investigated in each OAI incidence cohort, OAI progression cohort, CHECK incidence cohort, and CHECK progression cohort.

In our secondary outcomes, we defined the end-stage hip OA as having an overall grade of 3 or 4, or undergoing total hip replacement (THR). The incidence of end-stage hip OA was identified in participants who developed these criteria at the 4-to-5-year follow-up while not having it at baseline. This outcome was investigated across all four cohorts: OAI incidence, OAI progression, CHECK incidence, and CHECK progression.

Symptomatic hip OA was determined by the presence of hip pain alongside having a radiographic hip OA (i.e., overall grade 2 or more). In the OAI cohort, hip pain was identified if participants reported experiencing pain, aching, or stiffness in the hip at any time in the 12 months preceding the visit (baseline or 4-year follow-up). In the CHECK cohort, hip pain was assessed during clinical evaluations during the visit (baseline or 5-year follow-up). The incidence of symptomatic hip OA was defined as cases present at the 4-to-5-year follow-up but not at baseline. Conversely, resolution of symptomatic hip OA was marked when these symptoms were absent at the 4-to-5-year follow-up, despite being present at baseline. The incidence and resolution of symptomatic hip OA were investigated in all four study cohorts. The resolution was studied only in progression cohorts of OAI and CHECK, as these participants had symptoms at baseline, enabling an assessment of their subsequent resolution.

Additionally, we conducted an analysis focusing on individuals with baseline hip pain across all cohorts to explore the relationship between long-term NSAID use, joint pain, and structural changes in hip OA.

### 2.4 Selection of participants and hips

**Fig. 1:**
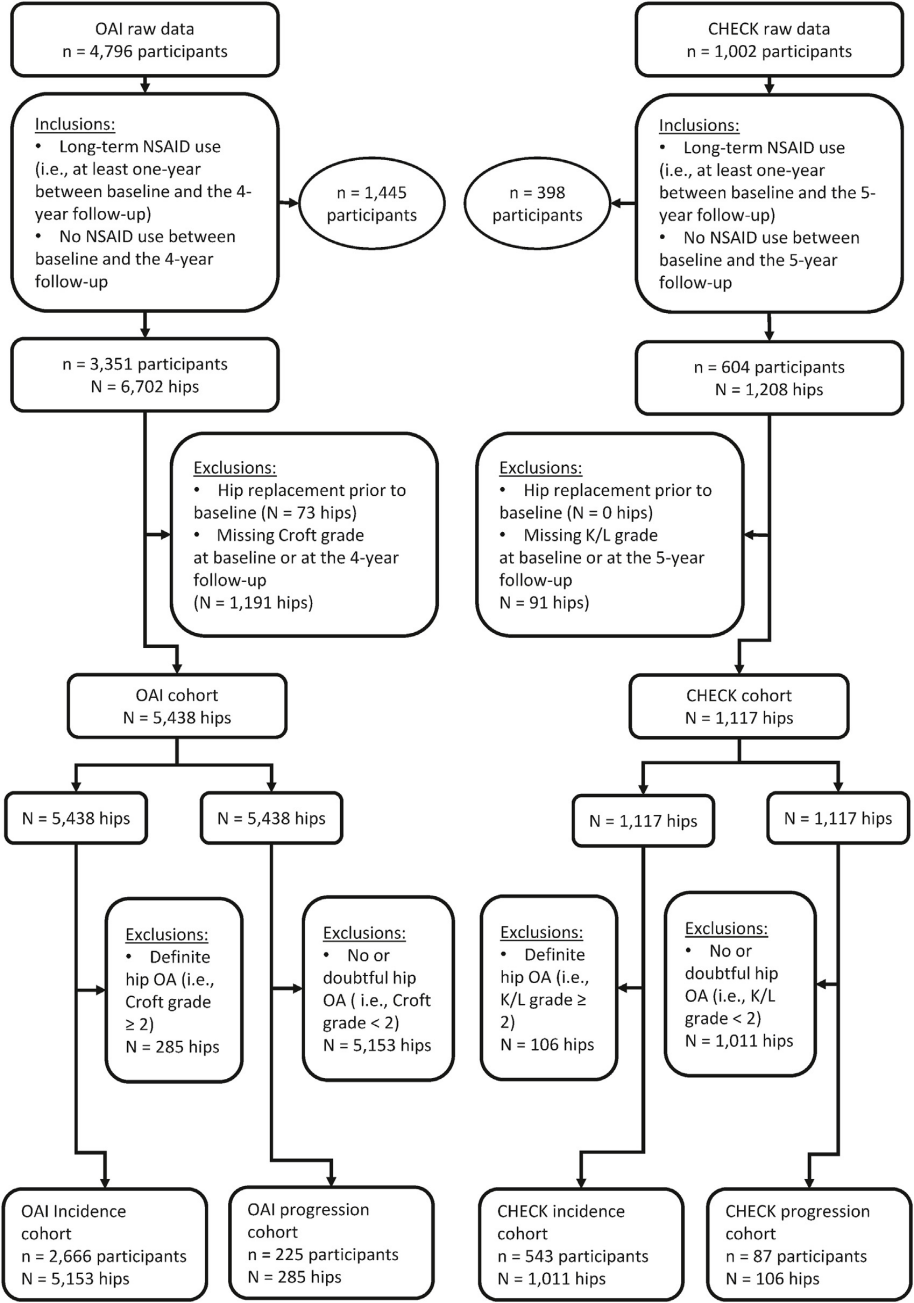
Selection of hips for the investigations of the outcomes. CHECK: Cohort Hip and Cohort Knee; K/L: Kellgren Lawrence, NSAIDs: Non-Steroidal Anti-Inflammatory Drugs; OA: Osteoarthritis; OAI: Osteoarthritis Initiative.

We applied selection criteria at participant and hip levels in the OAI and CHECK study cohorts. Firstly, at the participant level, we only included participants who had long-term NSAID use (i.e., NSAID use at least one year between baseline and the 4-to-5-year follow-up), and had no NSAID use (i.e., no NSAID use between baseline and the 4-to-5-year follow-up). Secondly, at the hip level, we excluded hips that had been replaced prior to baseline and had missing overall grade data at baseline or the 4-to-5-year follow-up (Fig. 1). The remaining hips were sorted into four groups that formed the basis of four study cohorts. The OAI incidence cohort and CHECK incidence cohort consisted of only the hips without radiographic hip OA. The OAI progression cohort and CHECK progression cohort consisted of only the hips with radiographic hip OA (Fig. 1).

### 2.5 Statistical analyses

We used generalized estimating equations with a logistic link function (i.e., logistic regression with clustering of both hips within individuals) [26] to investigate the association between long-term use of NSAIDs and outcomes between baseline and the 4-to-5-year follow-up. All analyses were adjusted for sex and baseline values of age, body mass index (BMI), and comorbidity status (binary as having no comorbidity or at least one comorbidity).

In our analysis, we treated the duration of NSAID use as a continuous variable. Additionally, we performed a sensitivity analysis categorizing NSAID use into cumulative timeframes: 1-year or more, 2-year or more, 3-year or more, and 4-year or more for all cohorts, with an additional 5-year for the CHECK cohorts. In this sensitivity analysis, each cumulative category of NSAID use was compared against the group of NSAID non-users. In the sensitivity analysis, we did not include the degeneration of cysts in acetabular subchondral; sclerosis in femoral subchondral; and deformity in femoral head as there were none or insufficient events for those individual radiographic features.

For all the statistical analyses, STATA/BE 17.0 for Windows (64-bit ×86-64) software was used. We set the threshold for statistical significance as a two-tailed *P* value of less than 0.05 for all statistical analyses.

## 3 Results

### 3.1 Baseline characteristics of the OAI and CHECK study cohorts

The OAI incidence cohorts comprised 5153 hips, and the CHECK incidence cohorts included 1011 hips. In contrast, the progression cohorts had fewer hips, with 285 in OAI and 106 in CHECK (Table 1). Participants in the OAI cohorts were generally older, less frequently female, heavier, and more frequently free of comorbidities than those in the CHECK cohorts. There were no hips with an overall grade of 3 or 4 in the OAI progression cohort, only 7 hips with an overall grade of 3, and none with an overall grade of 4 in the CHECK progression cohort. Approximately half of the participants reported pain among those who used NSAIDs for at least one year or more during the 4-to-5-year follow-up.

**Table 1:** Baseline characteristics of participants in the OAI incidence cohort, OAI progression cohort, CHECK incidence cohort, and CHECK progression cohort, stratified by at least one year use of non-steroidal anti-inflammatory drugs during 4–5 years follow-up.

Characteristics	OAI cohort				CHECK cohort			
	OAI incidence cohort		OAI progression cohort		CHECK incidence cohort		CHECK progression cohort	
	NSAID users ≥1-year	NSAID non-users	NSAID users ≥1-year	NSAID non-users	NSAID users ≥1-year	NSAID non-users	NSAID users ≥1-year	NSAID non-users
Hips (Participants)	N ​= ​892 (n ​= ​465)	N ​= ​4261 (n ​= ​2201)	N ​= ​67 (n ​= ​51)	N ​= ​218 (n ​= ​174)	N ​= ​251 (n ​= ​137)	N ​= ​760 (n ​= ​406)	N ​= ​40 (n ​= ​30)	N ​= ​66 (n ​= ​57)
Age, years	61.0 ​**±** ​8.5	60.9 ​**±** ​9.1	62.0 ​**±** ​8.8	64.9 ​**±** ​8.8	54.6 ​**±** ​4.9	56.1 ​**±** ​5.2	57.8 ​**±** ​4.3	57.2 ​**±** ​4.7
Sex								
Male	335 (37.6)	1880 (44.1)	36 (53.7)	142 (65.1)	26 (10.4)	142 (18.7)	10 (25.0)	28 (42.4)
Female	557 (62.4)	2381 (55.9)	31 (46.3)	76 (34.9)	225 (89.6)	618 (81.3)	30 (75.0)	38 (57.6)
Body mass index, kg/m^2^	29.4 ​**±** ​5.0	27.8 ​**±** ​4.5	28.8 ​**±** ​3.9	28.0 ​**±** ​4.0	27.1 ​**±** ​4.3	26.0 ​**±** ​4.0	26.5 ​**±** ​4.3	26.5 ​**±** ​3.8
Severity of radiographic hip OA∗ (overall grade score)								
Grade 0	772 (86.6)	3728 (87.5)	0 (0.0)	0 (0.0)	209 (83.3)	553 (72.8)	0 (0.0)	0 (0.0)
Grade 1	120 (13.4)	533 (12.5)	0 (0.0)	0 (0.0)	42 (16.7)	207 (27.2)	0 (0.0)	0 (0.0)
Grade 2	0 (0.0	0 (0.0	67 (100.0)	218 (100.0)	0 (0.0)	0 (0.0)	38 (95.0)	61 (92.4)
Grade 3	0 (0.0)	0 (0.0)	0 (0.0)	0 (0.0)	0 (0.0)	0 (0.0)	2 (5.0)	5 (7.6)
Grade 4	0 (0.0)	0 (0.0)	0 (0.0)	0 (0.0)	0 (0.0)	0 (0.0)	0 (0.0)	0 (0.0)
Comorbidity score								
0 (None)	665 (74.6)	3364 (79.0)	45 (67.2)	167 (76.6)	40 (15.9)	221 (29.1)	6 (15.0)	23 (34.9)
1 or more	217 (24.3)	869 (20.3)	22 (32.8)	50 (22.9)	209 (83.3)	539 (70.9)	32 (80.0)	43 (65.1)
Missing	10 (1.1)	28 (0.7)	0 (0.0)	1 (0.5)	2 (0.8)	0 (0.0)	2 (5.0)	0 (0.0)
Presence of hip pain								
None	427 (47.9)	2707 (63.5)	30 (44.8)	115 (52.8)	135 (53.8)	508 (66.8)	20 (50.0)	31 (47.0)
Yes	464 (52.0)	1543 (36.2)	37 (55.2)	103 (47.2)	116 (46.2)	252 (33.2)	20 (50.0)	35 (53.0)
Missing	1 (0.1)	11 (0.3)	0 (0.0)	0 (0.0)	0 (0.0)	0 (0.0)	0 (0.0)	0 (0.0)

Data are presented as mean ​± ​standard deviation or count (percentage). The two incidence cohorts consisted only of hips without radiographic hip OA (i.e., an overall grade score of <2) at baseline. The two progression cohorts consisted of only hips with radiographic hip OA (i.e., an overall grade score of ≥2) at baseline. ∗Assessed by K/L grade in the OAI study and modified Croft grade in the CHECK study. CHECK: Cohort of Hip and Cohort of Knee; CI: Confidence interval; K/L: Kellgren/Lawrence; NSAIDs: Non-Steroidal Anti-Inflammatory Drugs; OA: Osteoarthritis; OAI: Osteoarthritis Initiative.

### 3.2 Primary outcomes

#### 3.2.1 Incidence and progression of the radiographic hip OA over 4–5 years

No association was found between NSAID use and the incidence of radiographic hip OA in any cohort (Table 2). This null association was also observed in participants with baseline hip pain. In the OAI progression cohort, no hips showed progression of radiographic hip OA over 4–5 years, and only four hips in the CHECK progression cohort exhibited progression. No association was observed between NSAID use and progression of radiographic hip OA in the CHECK progression cohort, including in participants with baseline hip pain.

**Table 2:** Association of use of NSAIDs and incidence and progression of radiographic hip OA, incidence of end-stage hip OA, and incidence and resolution of symptomatic hip OA.

Outcome	Incidence Cohorts		Progression Cohorts	
	OAI	CHECK	OAI	CHECK
All participants				
Hips NSAID users ≥ 1-year NSAID non-users	N=5,153 N=892 (17.3) N=4,261 (82.7)	N=1,011 N=251 (24.8) N=760 (75.2)	N=285 N=67 (23.5) N=218 (76.5)	N=106 N=40 (37.7) N=66 (62.3)
Participants with hip pain at baseline				
Hips NSAID users ≥ 1-year NSAID non-users	N=2,007 N=464 (23.1) N=1,543 (76.9)	N=368 N=116 (31.5) N=252 (68.5)	N=140 N=37 (26.4) N=103 (73.6)	N=55 N=20 (36.4) N=35 (63.6)
**Incidence of radiographic hip OA**				
All participants				
Events NSAID users ≥ 1-year NSAID non-users	13 (1.5) 66 (1.5)	26 (10.3) 94 (12.4)	N/A	N/A
Odds ratio (95% CI)	0.98 (0.79 to 1.22)	0.88 (0.72 to 1.08)	N/A	N/A
Participants with hip pain at baseline				
Events NSAID users ≥ 1-year NSAID non-users	10 (2.2) 29 (1.9)	17 (14.7) 36 (14.3)	N/A	N/A
Odds ratio (95% CI)	1.05 (0.81 to 1.34)	0.89 (0.68 to 1.18)	N/A	N/A
**Progression of radiographic hip OA**				
All participants				
Events NSAID users ≥ 1-year NSAID non-users	N/A	N/A	0 (0.0) 0 (0.0)	2 (5.0) 2 (3.0)
Odds ratio (95% CI)	N/A	N/A	No observation	0.81 (0.28 to 2.37))
Participants with hip pain at baseline				
Events NSAID users ≥ 1-year NSAID non-users	N/A	N/A	0 (0.0) 0 (0.0)	1 (5.0) 2 (5.7)
Odds ratio (95% CI)	N/A	N/A	No observation	0.64 (0.12 to 3.37))
**Incidence of end-stage hip OA**				
All participants				
Events NSAID users ≥ 1-year NSAID non-users	7 (0.8) 6 (0.1))	2 (0.8) 10 (1.3)	12 (17.9) 17 (7.8)	6 (15.0) 13 (19.7)
Odds ratio (95% CI)	**1.59 (1.16 to 2.19)**	0.94 (0.56 to 1.58)	1.34 (0.99 to 1.81)	1.09 (0.73 to 1.63))
Participants with hip pain at baseline				
Events NSAID users ≥ 1-year NSAID non-users	6 (1.3) 3 (0.2)	2 (1.7) 7 (2.8)	9 (24.3) 14 (13.6)	5 (25.0) 13 (37.1)
Odds ratio (95% CI)	**1.64 (1.12 to 2.39)**	1.06 (0.63 to 1.77)	1.24 (0.87 to 1.75)	1.03 (0.67 to 1.60))
**Incidence of symptomatic hip OA**				
All participants				
Events NSAID users ≥ 1-year NSAID non-users	8 (0.9) 35 (0.8)	14 (5.6) 34 (4.5)	13 (19.4) 36 (16.5)	6 (15.0) 7 (10.6)
Odds ratio (95% CI)	0.94 (0.70 to 1.25)	0.98 (0.76 to 1.26)	1.05 (0.71 to 1.54)	0.85 (0.47 to 1.53))
Participants with hip pain at baseline				
Events NSAID users ≥ 1-year NSAID non-users	7 (1.5) 19 (1.2)	10 (8.6) 20 (7.9)	N/A	N/A
Odds ratio (95% CI)	1.04 (0.77 to 1.41)	1.04 (0.77 to 1.40)	N/A	N/A
**Resolution of symptomatic hip OA**				
All participants				
Events NSAID users ≥ 1-year NSAID non-users	N/A	N/A	18 (26.9) 50 (22.9)	6 (15.0) 13 (19.7)
Odds ratio (95% CI)	N/A	N/A	0.99 (0.73 to 1.34)	0.91 (0.56 to 1.46))
Participants with hip pain at baseline				
Events NSAID users ≥ 1-year NSAID non-users	N/A	N/A	18 (48.7) 50 (48.5)	6 (30.0 13 (37.1))
Odds ratio (95% CI)	N/A	N/A	0.99 (0.73 to 1.34)	0.91 (0.56 to 1.46))

The brackets show the percentage of events among the respective groups of NSAID users ≥ 1-year and NSAID non-users. The estimations were adjusted for sex and baseline values of age, body mass index, and comorbidity status at baseline. CHECK: of Hip and Cohort of Knee; CI: Confidence interval; NSAIDs: Non-Steroidal Anti-Inflammatory Drugs; OA: Osteoarthritis; OAI: Osteoarthritis Initiative.

#### 3.2.2 Degeneration of individual radiographic features of the hip OA over 4–5 years

Across all four cohorts, there was no evidence of an association between NSAID use and the degeneration of any of the nine individual radiographic features of hip OA, including participants with hip pain at baseline, except for sclerosis in the femoral subchondral in the OAI incidence cohort (Odds Ratio (OR): 2.57, 95 ​% Confidence Intervals (CI) 1.32 to 5.01) (Table 3). This finding was based on three hips (two for NSAID users ≥1 year and one for NSAID non-users) (Table 3). In participants with hip pain at baseline in the OAI incidence cohort, only one event of sclerosis in femoral subchondral was observed, precluding the calculation of an association (Table 3).

**Table 3:** Association of use of NSAIDs and degeneration of individual radiographic hip OA outcomes.

Outcome	Incidence Cohorts		Progression Cohorts	
	OAI	CHECK	OAI	CHECK
All participants				
Hips	N ​= ​5153	N ​= ​1011	N ​= ​285	N ​= ​106
NSAID users ≥ 1-year	N ​= ​892 (17.3)	N ​= ​251 (24.8)	N ​= ​67 (23.5)	N ​= ​40 (37.7)
NSAID non-users	N ​= ​4261 (82.7)	N ​= ​760 (75.2)	N ​= ​218 (76.5)	N ​= ​66 (62.3)
Participants with hip pain at baseline				
Hips	N ​= ​2007	N ​= ​368	N ​= ​140	N ​= ​55
NSAID users ≥ 1-year	N ​= ​464 (23.1)	N ​= ​116 (31.5)	N ​= ​37 (26.4)	N ​= ​20 (36.4)
NSAID non-users	N ​= ​1543 (76.9)	N ​= ​252 (68.5)	N ​= ​103 (73.6)	N ​= ​35 (63.6)
**Joint space narrowing lateral**				
All participants				
Events				
NSAID users ≥ 1-year	9 (1.0)	17 (6.8)	7 (10.5)	3 (7.5)
NSAID non-users	72 (1.7)	66 (8.7)	26 (11.9)	4 (6.1)
Odds ratio (95 ​% CI)	0.88 (0.68–1.14)	0.95 (0.76–1.18)	0.90 (0.59–1.36)	1.20 (0.69–2.11)
Participants with hip pain at baseline				
Events				
NSAID users ≥ 1-year	6 (1.3)	8 (6.9)	3 (8.1)	0 (0.0)
NSAID non-users	23 (1.5)	21 (8.3)	14 (13.6)	2 (5.7)
Odds ratio (95 ​% CI)	1.06 (0.79–1.42)	0.77 (0.51–1.17)	0.94 (0.55–1.59)	No observation
**Joint space narrowing medial**				
All participants				
Events				
NSAID users ≥ 1-year	35 (3.9)	48 (19.1)	9 (13.4)	4 (10.0)
NSAID non-users	119 (2.8)	134 (17.6)	32 (14.7)	6 (9.1)
Odds ratio (95 ​% CI)	1.10 (0.95–1.27)	1.04 (0.89–1.21)	0.90 (0.62–1.31)	1.08 (0.56–2.06)
Participants with hip pain at baseline				
Events				
NSAID users ≥ 1-year	20 (4.3)	25 (21.6)	5 (13.5)	4 (20.0)
NSAID non-users	46 (3.0)	43 (17.1)	12 (11.7)	5 (14.3)
Odds ratio (95 ​% CI)	1.18 (0.99–1.42)	1.17 (0.95–1.44)	1.07 (0.69–1.65)	1.30 (0.63–2.70)
**Osteophytes acetabular superior**				
All participants				
Events				
NSAID users ≥ 1-year	16 (1.8)	53 (21.1)	5 (7.5)	12 (30.0)
NSAID non-users	56 (1.3)	217 (28.6)	16 (7.3)	12 (18.2)
Odds ratio (95 ​% CI)	1.15 (0.95–1.40)	0.88 (0.76–1.01)	0.83 (0.48–1.45)	1.24 (0.88–1.74)
Participants with hip pain at baseline				
Events				
NSAID users ≥ 1-year	10 (2.2)	26 (22.4)	3 (8.1)	4 (20.0)
NSAID non-users	28 (1.8)	77 (30.6)	8 (7.8)	7 (20.0)
Odds ratio (95 ​% CI)	1.03 (0.78–1.36)	0.94 (0.77–1.15)	0.83 (0.42–1.64)	1.08 (0.62–1.87)
**Osteophytes acetabular inferior**				
All participants				
Events				
NSAID users ≥ 1-year	3 (0.3)	15 (6.0)	2 (3.0)	9 (22.5)
NSAID non-users	19 (0.5)	65 (8.6)	11 (5.1)	3 (4.6)
Odds ratio (95 ​% CI)	0.64 (0.29–1.41)	0.94 (0.75–1.18)	1.07 (0.66–1.73)	1.40 (0.95–2.06)
Participants with hip pain at baseline				
Events				
NSAID users ≥ 1-year	0 (0.0)	9 (7.8)	1 (2.7)	5 (25.0)
NSAID non-users	9 (0.6)	20 (7.9)	6 (5.8)	3 (8.6)
Odds ratio (95 ​% CI)	No observation	1.03 (0.75–1.44)	0.95 (0.47–1.92)	0.92 (0.50–1.69)
**Osteophytes femoral superior**				
All participants				
Events				
NSAID users ≥ 1-year	18 (2.0)	68 (27.1)	8 (11.9)	14 (35.0)
NSAID non-users	72 (1.7)	184 (24.2)	21 (9.6)	15 (22.7)
Odds ratio (95 ​% CI)	0.98 (0.80–1.21)	1.08 (0.95–1.23)	1.03 (0.70–1.53)	1.32 (0.98–1.78)
Participants with hip pain at baseline				
Events				
NSAID users ≥ 1-year	9 (1.9)	34 (29.3)	4 (10.8)	8 (40.0)
NSAID non-users	33 (2.1)	60 (23.8)	11 (10.7)	8 (22.9)
Odds ratio (95 ​% CI)	0.83 (0.60–1.16)	1.11 (0.92–1.34)	0.85 (0.47–1.54)	1.44 (0.85–2.46)
**Osteophytes femoral inferior**				
All participants				
Events				
NSAID users ≥ 1-year	5 (0.6)	37 (14.7)	2 (3.0)	8 (20.0)
NSAID non-users	16 (0.4)	93 (12.2)	22 (10.1)	9 (13.6)
Odds ratio (95 ​% CI)	1.07 (0.74–1.54)	0.99 (0.84–1.17)	0.72 (0.39–1.34)	1.22 (0.87–1.71)
Participants with hip pain at baseline				
Events				
NSAID users ≥ 1-year	2 (0.4)	19 (16.4)	2 (5.4)	5 (25.0)
NSAID non-users	6 (0.4)	35 (13.9)	7 6.8)	4 (11.4)
Odds ratio (95 ​% CI)	1.04 (0.60–1.79)	0.91 (0.69–1.19)	1.00 (0.53–1.88)	1.23 (0.72–2.12)
**Total osteophytes score ≥ 2**				
All participants				
Events				
NSAID users ≥ 1-year	8 (0.9)	38 (15.1)	7 (10.5)	10 (25.0)
NSAID non-users	26 (0.6)	136 (17.9)	17 (7.8)	10 (15.2)
Odds ratio (95 ​% CI)	0.93 (0.64–1.33)	0.98 (0.84–1.15)	1.08 (0.72–1.61)	1.10 (0.78–1.55)
Participants with hip pain at baseline				
Events				
NSAID users ≥ 1-year	5 (1.1)	20 (17.2)	4 (10.8)	3 (15.0)
NSAID non-users	13 (0.8)	49 (19.4)	8 (7.8)	8 (22.9)
Odds ratio (95 ​% CI)	0.84 (0.51–1.41)	0.98 (0.77–1.25)	1.07 (0.65–1.77)	0.84 (0.47–1.50)
**Subchondral cysts acetabular**				
All participants				
Events				
NSAID users ≥ 1-year	1 (0.1)	0 (0.0)	1 (1.5)	0 (0.0)
NSAID non-users	6 (0.1)	2 (0.3)	6 (2.8)	1 (1.5)
Odds ratio (95 ​% CI)	1.01 (0.51–2.03)	No observation	1.12 (0.59–2.13)	No observation
Participants with hip pain at baseline				
Events				
NSAID users ≥ 1-year	1 (0.2)	0 (0.0)	1 (2.7)	0 (0.0)
NSAID non-users	2 (0.1)	1 (0.4)	3 (2.9)	0 (0.0)
Odds ratio (95 ​% CI)	1.27 (0.61–2.66)	No observation	1.33 (0.68–2.58)	No observation
**Subchondral sclerosis femoral**				
All participants				
Events				
NSAID users ≥ 1-year	2 (0.2)	0 (0.0)	1 (1.5)	1 (2.5)
NSAID non-users	1 (0.0)	5 (0.7)	7 (3.2)	3 (4.6)
Odds ratio (95 ​% CI)	**2.57 (1.32 to 5.01)**	No observation)	0.56 (0.14–2.27)	0.25 (0.02–2.89)
Participants with hip pain at baseline				
Events				
NSAID users ≥ 1-year	1 (0.2)	0 (0.0)	1 (2.7)	1 (5.0)
NSAID non-users	0 (0.0)	4 (1.6)	2 (1.9)	2 (5.7)
Odds ratio (95 ​% CI)	No observation	No observation	0.67 (0.11–3.89)	0.22 (0.00–14.62)
**Femoral head deformity**				
All participants				
Events				
NSAID users ≥ 1-year	1 (0.1)	14 (5.6)	0 (0.0)	2 (5.0)
NSAID non-users	1 (0.0)	31 (4.1)	2 (0.9)	0 (0.0)
Odds ratio (95 ​% CI)	1.49 (0.68–3.29)	1.09 (0.81–1.47)	No observation	No observation
Participants with hip pain at baseline				
Events				
NSAID users ≥ 1-year	1 (0.2)	7 (6.0)	0 (0.0)	1 (5.0)
NSAID non-users	1 (0.1)	9 (3.6)	1 (1.0)	0 (0.0)
Odds ratio (95 ​% CI)	1.49 (0.68–3.29)	1.29 (0.89–1.87)	No observation	No observation

The numbers in brackets show the percentage of events among the respective groups of NSAID users ≥ 1-year and NSAID non-users. The estimations were adjusted for sex and baseline values of age; body mass index, comorbidity status at baseline. CHECK: Cohort of Hip and Cohort of Knee; CI: Confidence interval; NSAIDs: Non-Steroidal Anti-Inflammatory Drugs; OA: Osteoarthritis; OAI: Osteoarthritis Initiative.

Generally, few events were noted for cysts in acetabular subchondral, sclerosis in femoral subchondral, and deformity in femoral head across all cohorts (Table 3).

### 3.3 Secondary outcomes

#### 3.3.1 Incidence of end-stage hip OA over 4–5 years

While no statistically significant association was found between NSAID use and the incidence of end-stage hip OA in the OAI progression, CHECK incidence, and CHECK progression cohorts, an association was observed in the OAI incidence cohort (OR: 1.59, 95 ​% CI 1.16 to 2.19) (Table 2). This association was also present among the participants with hip pain at baseline in the OAI incidence cohort (OR: 1.64, 95 ​% CI 1.12 to 2.39) (Table 2) but not in the other three cohorts.

#### 3.3.2 Incidence of symptomatic hip OA and resolution of symptomatic hip OA over 4–5 years

No statistically significant association was observed between NSAID use and either the incidence or resolution of symptomatic hip OA over 4–5 years in any of the cohorts.

### 3.4 Sensitivity analyses

The sensitivity analyses yielded results similar to or consistent with those from the primary analyses (Tables S1 and S2).

## 4 Discussion

This study demonstrated that long-term use of NSAIDs was not associated with increased odds of incidence or progression of radiographic hip OA, nor with the degeneration of individual radiographic features of the hip, in either the OAI or CHECK cohorts over a period of 4–5 years. Similarly, no association was found regarding the incidence of end-stage hip OA, as well as the incidence and resolution of symptomatic hip OA. These findings suggest that NSAIDs may not significantly affect the hip joint structure over this time frame.

Our findings did present a few anomalies in the context of null associations. First, in the OAI incidence cohort, the association between NSAID use and sclerosis in the femoral subchondral suggested a possible detrimental effect. However, this was based on only three hips and not observed in the CHECK incidence cohort or the other two progression cohorts, indicating that this finding may be an outlier. Second, an association between NSAID use and the incidence of end-stage hip OA was observed in the OAI incidence cohort. In this cohort, end-stage hip OA was determined based on THR, as no hips had a K/L score of 3 or greater. While THR is a marker for end-stage hip OA, its occurrence can be influenced by external factors such as education, readiness for surgery, income, and health insurance [27,28]. Therefore, these THR cases may not directly reflect the progression of end-stage hip OA. Additionally, the absence of this association in the other three cohorts suggests that it might be an isolated occurrence.

Previous studies [[14], [15], [16], [17]] on the association between NSAID use and structural defects in hip OA have shown mixed results, ranging from no evidence of disease progression to suggestions of accelerated progression with specific NSAIDs. The first study, an observational study, suggested that diclofenac might induce accelerated progression of radiographic hip OA [14]. The second study, a 12-month cohort study, found no evidence of disease progression with 12-month celecoxib treatment, although there was worsening in JSN [15]. The third study, a two-year case-control study, compared NSAID use in subjects with hip OA who underwent hip replacement versus those treated medically. The study could not conclude any positive or negative effect of NSAID use on hip joint cartilage [16]. The fourth study, randomized the patients with hip OA with the treatment of indomethacin (a strong prostaglandin inhibitor, classified as an NSAID) or azapropazone (a weaker prostaglandin inhibitor). Patients treated with indomethacin underwent hip replacements sooner than the azapropazone group [17], suggesting the negative effect of indomethacin on the hip joint.

In contrast to our null findings in hip OA, existing research [14,[29], [30], [31]], suggests a link between long-term NSAID use and structural changes in knee OA, likely due to the pain-masking effect of NSAIDs leading to increased joint stress. This disparity may be attributed to anatomical differences between the hip and knee joints. The ball-and-socket structure of the hip joint distributes stress more uniformly, possibly reducing the impact of NSAID-induced activity on joint deterioration. In contrast, the hinge-like architecture of the knee joint focuses stress more intensely, increasing susceptibility to damage. This highlights the need for joint-specific strategies in OA research and NSAID management, emphasizing the importance of understanding anatomical differences in developing effective treatments.

While our results suggest that long-term NSAID usage may not significantly affect hip joint structure over 4–5 years, the potential serious side effects of NSAIDs, such as gastrointestinal complications [6], cardiovascular disease [7], and renal failure [8], warrant caution. Healthcare providers should prioritize short-term NSAID use for symptom relief and consider alternative management strategies for hip OA, like patient education, exercise, and weight management. These strategies align with established guidelines for hip OA management [[32], [33], [34]], enhancing patient outcomes while minimizing NSAID-related risks.

Our study has several limitations. First, we assumed continuous NSAID use between visits, which was supported by findings that the average duration of NSAID treatment for hip OA is around 16 months [13]. However, we lacked data to confirm or refute intermittent NSAID use. Similarly, we did not have information on the duration of NSAID use prior to study entry for those already using NSAIDs at baseline. This limitation could contribute to our null findings for the association between NSAID use and the outcomes. Second, we observed reduced compliance in NSAID use each year, potentially affecting effect size. Third, the limited number of events, particularly in the progression cohorts, constraints our conclusions about NSAID use and hip OA progression. Despite this, our consistent findings of no evidence of degeneration in any of the nine individual structural features, including the sum of individual grades for osteophytes in all four locations, partly mitigate this limitation. Additionally, the results for specific individual radiographic features, such as subchondral cysts acetabular, subchondral sclerosis femoral, and femoral head deformity, may be less reliable due to the low occurrence of events during the 4-to-5-year follow-up period. Fourth, a key limitation is the lack of detailed information on the specific reasons for NSAID use in the OAI dataset, although some data from the CHECK dataset do indicate whether NSAIDs were used for knee or hip complaints. This gap may limit our understanding of the precise motivations behind NSAID use, particularly in differentiating whether it was for knee pain or hip pain. Additionally, including participants with hip and/or knee pain in our cohorts poses a question about the extent to which NSAID use for knee pain might have influenced our results. Nevertheless, the consistent observation of null associations in the general cohort and specifically in the subgroup with only hip pain suggests that our findings apply to individuals with hip pain, irrespective of concurrent knee pain. Such consistency indicates that the potential confounding impact of knee pain on NSAID use patterns does not significantly alter the applicability of our results to hip OA outcomes. Therefore, while the possibility of NSAID use for knee pain and the mixed nature of our cohorts present limitations, these factors do not substantially detract from the relevance of our findings for clinical practice in managing hip OA. Fifth, the limited sensitivity of radiographs compared to more advanced methods like MRI may have affected our ability to detect subtle changes. Further studies using advanced imaging techniques are necessary to explore the association between NSAID use and structural changes in hip OA. Lastly, the predominance of female and White participants in our cohorts limits the generalizability of our findings.

In conclusion, we found no association between long-term NSAID use and the incidence or progression of radiographic hip OA, nor with the degeneration of individual radiographic features over 4–5 years. Similarly, we found no association between the incidence of end-stage hip OA, as well as and the incidence and resolution of symptomatic hip OA.

## Contributions

Conception and design: ZS.

Statistical analysis: ZS.

Drafting the paper: ZS.

Critical review of the manuscript: ZS.

Final approval: ZS.

## Role of the funding sources

There was no funders for this study, therefore, the study is free of funder involvement in study design, data collection, data analysis, data interpretation, or writing of the report. The corresponding author had full access to all the data in the study and had final responsibility for the decision to submit for publication.

## Declaration of competing interest

ZS owns 50% of the shares in Zuman International Pty. Ltd., which receives royalties and other payments for educational resources and services in adult weight management and research methodology.
