# Supplementary material for: Investigation of the association of long-term NSAID use with radiographic hip osteoarthritis over four to five years: Data from the OAI and CHECK studies
Source: Osteoarthr Cartil Open. 2023 Dec 9;6(1):100427. doi: 10.1016/j.ocarto.2023.100427 (PMC10770760; doi:10.1016/j.ocarto.2023.100427)
Supplement: Multimedia component 1 [file mmc1.docx]

**SUPPLEMENTARY FILE**

**Investigation of the Association of Long-term NSAID Use with Radiographic Hip Osteoarthritis Over Four to Five Years: Data from the OAI and CHECK Studies**

**Table S1.** Association of long-term use of NSAIDs by categorizing NSAID use into cumulative timeframes and incidence and progression of radiographic hip OA, incidence of end-stage hip OA, incidence and resolution of symptomatic hip OA during 4 to 5 years follow-up (sensitivity analysis)

| Outcome | OAI | | | | | CHECK | | | | | | | |
| --- | --- | --- | --- | --- | --- | --- | --- | --- | --- | --- | --- | --- | --- |
|  | NSAID users  ≥ 1-year | NSAID users  ≥ 2-year | NSAID users  ≥ 3-year | NSAID users  ≥ 4-year | NSAID  non-users | NSAID users  ≥ 1-year | NSAID users  ≥ 2-year | | NSAID users  ≥ 3-year | | NSAID users  ≥ 4-year | | NSAID  non-users |
| **INCIDENCE COHORTS** | | | | | | | | | | | | | |
| All participants | N=892 | N=576 | N=415 | N=314 | N=4,261 | N=251 | N=140 | | N=90 | N=62 | | | N=760 |
| Participants with hip pain at baseline | N=464 | N=303 | N=219 | N=167 | N=1,543 | N=116 | N=67 | | N=38 | N=23 | | | N=252 |
| **Incidence of radiographic hip OA** | | | | | | | | | | | | | |
| All participants | | | | | | | | | | | | | |
| Events N (%) | 13  (1.5) | 8  (1.4) | 7  (1.7) | 5  (1.6) | 66  (1.6) | 26  (10.4) | 11  (7.9) | | 6  (6.7) | | 3  (4.8) | | 94  (12.4) |
| Odds ratio  (95% CI) | 0.95  (0.51-1.76) | 0.88  (0.41-1.88)) | 1.04  (0.46-2.33) | 0.95  (0.37-2.44) | 1  (reference) | 0.89  (0.53-1.50) | 0.65  (0.31-1.34) | | 0.56  (0.22-1.42) | | 0.42  (0.11-1.50) | | 1  (reference) |
| Participants with hip pain at baseline | | | | | | | | | | | | | |
| Events N (%) | 10  (2.2) | 6  (2.0) | 5  (2.3) | 3  (1.8) | 29  (1.9) | 17  (14.7) | 6  (9.0) | | 2  (5.3) | | 1  (4.4) | | 36  (14.3) |
| Odds ratio  (95% CI) | 1.27  (0.61-2.63) | 1.14  (0.47-2.78) | 1.28  (0.49-3.35) | 0.95  (0.29-3.16) | 1  (reference) | 1.20  (0.60-2.76) | 0.73  (0.28-1.91) | | 0.37  (0.08-1.71) | | 0.34  (0.04-2.70) | | 1  (reference) |
| **Incidence of end-stage hip OA** | | | | | | | | | | | | | |
| All participants | | | | | | | | | | | | | |
| Events N (%) | 7  (0.8) | 5  (0.9) | 5  (1.2) | 3  (1.0) | 6  (0.1) | 2  (0.8) | 1  (0.7) | | 1  (1.1) | | 1  (1.6) | | 10  (1.3) |
| Odds ratio  (95% CI) | **4.46**  **(1.45-13.79)** | **5.30**  **(1.55-18.08))** | **7.66**  **(2.24-26.23)** | **6.86**  **(1.63-28.92)** | 1  (reference) | 0.56  (0.12-2.71) | 0.51  (0.06-4.23) | | 0.85  (0.10-6.94) | | 1.33  (0.16-10.99) | | 1  (reference) |
| Participants with hip pain at baseline | | | | | | | | | | | | | |
| Events N (%) | 6  (1.3) | 4  (1.3) | 4  (1.8) | 2  (1.2) | 3  (0.2) | 2  (1.7) | 1  (1.5) | | 1  (2.6) | | 1  (4.4) | | 7  (2.8) |
| Odds ratio  (95% CI) | **6.18**  **(1.51-25.29)** | **6.54**  **(1.43-29.93)** | **9.66**  **(2.09-44.59)** | **7.37**  **(1.18-46.15)** | 1  (reference) | 0.82  (0.16-4.29) | 0.79  (0.09-6.98) | | 1.28  (0.14-11.40) | | 2.14  (0.24-19.41) | | 1  (reference) |
| **Incidence of symptomatic hip OA** | | | | | | | | | | | | | |
| All participants | | | | | | | | | | | | | |
| Events N (%) | 8  (0.9) | 4  (0.7) | 3  (0.7) | 2  (0.6) | 35  (0.8) | 14  (5.6) | 7  (5.0) | | 3  (3.3) | | 1  (1.6) | | 34  (4.5) |
| Odds ratio  (95% CI) | 1.02  (0.47-2.24) | 0.76  (0.27-2.16) | 0.77  (0.23-2.52) | 0.65  (0.15-2.75) | 1  (reference) | 1.25  (0.64-2.46) | 1.13  (0.47-2.71) | | 0.77  (0.22-2.62) | | 0.41  (0.05-3.11) | | 1  (reference) |
| Participants with hip pain at baseline | | | | | | | | | | | | | |
| Events N (%) | 7  (1.5) | 4  (1.3) | 3  (1.4) | 2  (1.2) | 19  (1.2) | 10  (8.6) | 6  (9.0) | | 2  (5.3) | | 1  (4.4) | | 20  (7.9) |
| Odds ratio  (95% CI) | 1.35  (0.56-3.25) | 1.15  (0.39-3.43) | 1.15  (0.33-3.95) | 0.95  (0.22-4.17) | 1  (reference) | 1.35  (0.59-3.07) | 1.52  (0.56-4.15) | | 0.80  (0.18-3.65) | | 0.76  (0.09-6.11) | | 1  (reference) |
| **PROGRESSION COHORTS** | | | | | | | | | | | | | |
| All participants | N=67 | N=32 | N=22 | N=18 | N=218 | N=40 | N=23 | N=12 | | | N=10 | N=66 | |
| Participants with hip pain at baseline | N=37 | N=19 | N=14 | N=11 | N=103 | N=20 | N=13 | N=7 | | | N=5 | N=35 | |
| **Progression of radiographic hip OA** | | | | | | | | | | | | | |
| All participants | | | | | | | | | | | | | |
| Events N (%) | 0  (0.0) | 0  (0.0) | 0  (0.0) | 0  (0.0) | 0  (0.0) | 2  (5.0) | 5  (21.7) | | 0  (0.0) | | 0  (0.0) | | 2  (3.0) |
| Odds ratio  (95% CI) | No observation | No observation | No observation | No observation | 1  (reference) | 2.1  (0.22-19.71) | 0.19  (0.00-14.35) | | No observation | | No observation | | 1  (reference) |
| Participants with hip pain at baseline | | | | | | | | | | | | | |
| Events N (%) | 0  (0.0) | 0  (0.0) | 0  (0.0) | 0  (0.0) | 0  (0.0) | 1  (5.0) | 0  (0.0) | | 0  (0.0) | | 0  (0.0) | | 2  (5.7) |
| Odds ratio  (95% CI) | No observation | No observation | No observation | No observation | 1  (reference) | 1.39  (0.61-31.76) | No observation | | No observation | | No observation | | 1  (reference) |
| **Incidence of end-stage hip OA** | | | | | | | | | | | | | |
| All participants | | | | | | | | | | | | | |
| Events N (%) | 12  (17.9) | 6  (18.8) | 5  (22.7) | 4  (22.2) | 17  (7.8) | 6  (15.0) | 5  (21.7) | | 2  (16.7) | | 2  (20.0) | | 13  (19.7) |
| Odds ratio  (95% CI) | **2.50**  **(1.06-5.87)** | 2.66  (0.89-7.96) | 3.20  (0.94-10.96) | 3.02  (0.82-11.13) | 1  (reference) | 0.60  (0.16-2.26) | 1.20  (0.28-5.03) | | 1.14  (0.14-9.24) | | 0.96  (0.08-12.19) | | 1  (reference) |
| Participants with hip pain at baseline | | | | | | | | | | | | | |
| Events N (%) | 9  (24.3) | 4  (21.1) | 4  (28.6) | 3  (27.3) | 14  (13.6) | 5  (25.0) | 4  (30.8) | | 2  (28.6) | | 2  (40.0) | | 13  (37.1) |
| Odds ratio  (95% CI) | 2.02  (0.75-5.43) | 1.84  (0.49-6.84) | 3.01  (0.74-12.26) | 2.74  (0.60-12.44) | 1  (reference) | 0.41  (0.10-1.72) | 0.64  (0.12-3.38) | | 1.14  (0.14-9.24) | | 0.96  (0.08-12.19) | | 1  (reference) |
| **Incidence of symptomatic hip OA** | | | | | | | | | | | | | |
| All participants | | | | | | | | | | | | | |
| Events N (%) | 13  (19.4) | 4  (12.5) | 2  (9.1) | 1  (5.6) | 36  (16.5) | 6  (15.0) | 1  (4.4) | | 0  (0.0) | | 0  (0.0) | | 7  (10.6) |
| Odds ratio  (95% CI) | 1.93  (0.78-4.77**)** | 1.04  (0.28-3.92) | 0.77  (0.13-4.47) | 0.40  (0.04-3.75) | 1  (reference) | 2.53  (0.54-11.84) | 0.53  (0.05-5.57) | | No observation | | No observation | | 1  (reference) |
| Participants with hip pain at baseline | | | | | | | | | | | | | |
| Events N (%) | N/A | N/A | N/A | N/A | N/A | N/A | N/A | | N/A | | N/A | | N/A |
| Odds ratio  (95% CI) | N/A | N/A | N/A | N/A | N/A | N/A | N/A | | N/A | | N/A | | N/A |
| **Resolution of symptomatic hip OA** | | | | | | | | | | | | | |
| All participants | | | | | | | | | | | | | |
| Events N (%) | 18  (26.9) | 6  (18.8) | 5  (22.7) | 4  (22.2) | 50  (22.9) | 6  (15.0) | 3  (13.0) | | 2  (16.7) | | 2  (20.0) | | 13  (19.7) |
| Odds ratio  (95% CI) | 1.50  (0.65-3.43**)** | 0.73  (0.24-2.24) | 0.97  (0.27-3.43) | 0.87  (0.22-3.43) | 1  (reference) | 0.51  (0.11-2.24**)** | 0.47  (0.08-2.90) | | 0.38  (0.04-3.37) | | 1.37  (0.09-21.6) | | 1  (reference) |
| Participants with hip pain at baseline | | | | | | | | | | | | | |
| Events N (%) | 18  (48.7) | 6  (31.6) | 5  (35.7) | 4  (36.4) | 50  (48.5) | 6  (30.0) | 3  (23.1) | | 2  (28.6) | | 2  (40.0) | | 13  (37.1) |
| Odds ratio  (95% CI) | 1.50  (0.65-3.43**)** | 0.73  (0.24-2.24) | 0.97  (0.27-3.43) | 0.87  (0.22-3.43) | 1  (reference | 0.51  (0.11-2.24**)** | 0.47  (0.08-2.90) | | 0.38  (0.04-3.37) | | 1.37  (0.09-21.6) | | 1  (reference |

The analysis was adjusted for: age at baseline; sex; body mass index at baseline, and comorbidity score at baseline. CHECK: Cohort of Hip and Cohort of Knee; CI: Confidence interval; NSAIDs: Non-Steroidal Anti-Inflammatory Drugs; OA: Osteoarthritis; OAI: Osteoarthritis Initiative.

**Table S2.** Association of long-term use of NSAIDs by categorizing NSAID use into cumulative timeframes and degeneration of individual radiographic features of the hip, during 4 to 5 years follow-up (sensitivity analysis)

| Outcome | OAI | | | | | CHECK | | | | | |
| --- | --- | --- | --- | --- | --- | --- | --- | --- | --- | --- | --- |
|  | NSAID users  ≥ 1-year | NSAID users  ≥ 2-year | NSAID users  ≥ 3-year | NSAID users  ≥ 4-year | NSAID  non-users | NSAID users  ≥ 1-year | NSAID users  ≥ 2-year | NSAID users  ≥ 3-year | NSAID users  ≥ 4-year | NSAID  non-users |  |
| **INCIDENCE COHORTS** | | | | | | | | | | | |
| All participants | N=892 | N=576 | N=415 | N=314 | N=4,261 | N=251 | N=140 | N=90 | N=62 | N=760 |  |
| Participants with hip pain at baseline | N=464 | N=303 | N=219 | N=167 | N=1,543 | N=116 | N=67 | N=38 | N=23 | N=252 |  |
| **Joint space narrowing lateral** | | | | | | | | | | | |
| All participants | | | | | | | | | | | |
| Events N (%) | 9  (1.0) | 7  (1.2) | 4  (1.0) | 3  (1.0) | 72  (1.7) | 17  (6.8) | 11  (7.9) | 5  (5.6) | 3  (4.8) | 66  (8.7) |  |
| Odds ratio  (95% CI) | 0.64  (0.31-1.31) | 0.77  (0.35-1.72) | 0.62  (0.22-1.72) | 0.61  (0.19-1.98) | 1  (reference) | 0.90  (0.49-1.64) | 1.12  (0.54-2.34) | 0.77  (0.28-2.11) | 0.66  (0.19-2.36) | 1  (reference) |  |
| Participants with hip pain at baseline | | | | | | | | | | | |
| Events N (%) | 6  (1.3) | 5  (1.7) | 4  (1.8) | 3  (1.8) | 29  (1.9) | 17  (14.7) | 3  (4.5) | 0  (0.0) | 0  (0.0) | 21  (8.3) |  |
| Odds ratio  (95% CI) | 0.94  (0.38-2.33) | 1.21  (0.46-3.22) | 1.37  (0.47-4.01) | 1.36  (0.40-4.59) | 1  (reference) | 0.92  (0.38-2.24) | 0.67  (0.18-2.45) | 0.17  (0.01-2.91) | 0.28  (0.02-4.98) | 1  (reference) |  |
| **Joint space narrowing medial** | | | | | | | | | | | |
| All participants | | | | | | | | | | | |
| Events N (%) | 35  (3.9) | 19  (3.3) | 18  (4.3) | 14  (4.5) | 119  (2.8) | 48  (19.1) | 25  (17.9) | 20  (22.2) | 11  (17.7) | 134  (17.6) |  |
| Odds ratio  (95% CI) | 1.47  (0.96-2.25) | 1.16  (0.67-2.00) | 1.50  (0.86-2.64) | 1.89  (0.80-2.80) | 1  (reference) | 1.19  (0.77-1.84) | 1.06  (0.61-1.85) | 1.39  (0.75-2.59) | 1.06  (0.48-2.34) | 1  (reference) |  |
| Participants with hip pain at baseline | | | | | | | | | | | |
| Events N (%) | 20  (4.3) | 13  (4.3) | 12  (5.5) | 9  (5.4) | 46  (3.0) | 25  (21.6) | 15  (22.4) | 11  (29.0) | 5  (21.7) | 43  (17.1) |  |
| Odds ratio  (95% CI) | 1.70  (0.95-3.03) | 1.68  (0.85-3.13) | 2.09  (1.02-4.25) | 1.89  (0.84-4.24) | 1  (reference) | 1.49  (0.81-2.75) | 1.55  (0.76-3.17) | **2.32**  **(1.02-5.28)** | 1.76  (0.59-5.23) | 1  (reference) |  |
| **Total osteophytes score ≥ 2** | | | | | | | | | | | |
| All participants | | | | | | | | | | | |
| Events N (%) | 8  (0.9) | 2  (0.4) | 1  (0.2) | 1  (0.3) | 26  (0.6) | 38  (15.1) | 20  (14.3) | 14  (15.6) | 10  (16.1) | 136  (17.9) |  |
| Odds ratio  (95% CI) | 1.55  (0.66-3.65) | 0.57  (0.13-2.54) | 0.36  (0.04-2.98) | 0.45  (0.05-3.78) | 1  (reference) | 0.90  (0.80-1.97) | 0.90  (0.51-1.61) | 1.04  (0.53-2.04) | 0.99  (0.44-2.22) | 1  (reference) |  |
| Participants with hip pain at baseline | | | | | | | | | | | |
| Events N (%) | 5  (1.1) | 1  (0.3) | 0  (0.0) | 0  (0.0) | 13  (0.8) | 20  (17.2) | 9  (13.4) | 5  (13.2) | 2  (8.7) | 49  (19.4) |  |
| Odds ratio  (95% CI) | 1.47  (0.50-4.32) | 0.43  (0.06-3.29) | 0.27  (0.16-4.53) | 0.33  (0.02-5.62) | 1  (reference) | 1.06  (0.64-2.32) | 1.00  (0.44-2.31) | 1.13  (0.39-3.27) | 0.73  (0.16-3.46) | 1  (reference) |  |
| **Osteophytes acetabular superior** | | | | | | | | | | | |
| All participants | | | | | | | | | | | |
| Events N (%) | 16  (1.8) | 12  (2.1) | 10  (2.4) | 7  (2.2) | 56  (1.3) | 53  (21.1) | 25  (17.9) | 18  (20.0) | 15  (24.2) | 217  (28.6) |  |
| Odds ratio  (95% CI) | 1.38  (0.75-2.55) | 1.63  (0.83-3.20) | 1.81  (0.87-3.78) | 1.69  (0.72-3.94) | 1  (reference) | 0.69  (0.47-1.03) | 0.56  (0.33-0.95) | 0.65  (0.35-1.21) | 0.83  (0.42-1.65) | 1  (reference) |  |
| Participants with hip pain at baseline | | | | | | | | | | | |
| Events N (%) | 10  (2.2) | 6  (2.0) | 4  (1.8) | 2  (1.2) | 28  (1.8) | 26  (22.4) | 14  (20.9) | 10  (26.3) | 7  (30.4) | 77  (30.6) |  |
| Odds ratio  (95% CI) | 1.29  (0.59-2.80) | 1.22  (0.48-3.09) | 1.01  (0.32-3.26) | 0.67  (0.16-2.89) | 1  (reference) | 0.72  (0.41-1.27) | 0.71  (0.35-1.42) | 0.91  (0.40-2.09) | 1.29  (0.48-3.50) | 1  (reference) |  |
| **Osteophytes acetabular inferior** | | | | | | | | | | | |
| All participants | | | | | | | | | | | |
| Events N (%) | 3  (0.3) | 0  (0.0) | 0  (0.0) | 0  (0.0) | 19  (0.5) | 15  (6.0) | 9  (6.4) | 5  (5.6) | 3  (4.8) | 65  (8.6) |  |
| Odds ratio  (95% CI) | 0.76  (0.21-2.84) | 0.17  (0.01-2.89) | 0.24  (0.01-3.94) | 0.30  (0.02-5.80) | 1  (reference) | 0.78  (0.41-1.48) | 0.89  (0.41-1.95) | 0.76  (0.28-2.09) | 0.64  (0.18-2.31) | 1  (reference) |  |
| Participants with hip pain at baseline | | | | | | | | | | | |
| Events N (%) | 0  (0.0) | 0  (0.0) | 0  (0.0) | 0  (0.0) | 9  (0.6) | 9  (7.8) | 5  (7.5) | 2  (5.3) | 1  (4.4) | 20  (7.9) |  |
| Odds ratio  (95% CI) | 0.16  (0.01-2.79) | 0.25  (0.01-4.38) | 0.36  (0.02-6.17) | 0.47  (0.03-8.07) | 1  (reference) | 1.16  (0.47-2.86) | 1.50  (0.50-4.50) | 1.04  (0.22-4.92) | 0.89  (0.11-7.42) | 1  (reference) |  |
| **Osteophytes femoral superior** | | | | | | | | | | | |
| All participants | | | | | | | | | | | |
| Events N (%) | 18  (2.0) | 12  (2.1) | 8  (1.9) | 4  (1.3) | 72  (1.7) | 68  (27.1) | 37  (26.4) | 25  (27.8) | 18  (29.0) | 184  (24.2) |  |
| Odds ratio  (95% CI) | 1.09  (0.62-1.92) | 1.03  (0.52-2.04) | 0.83  (0.35-1.95) | 0.72  (0.26-2.01) | 1  (reference) | 1.25  (0.86-1.82) | 1.26  (0.79-2.01) | 1.31  (0.76-2.27) | 1.39  (0.73-2.63) | 1  (reference) |  |
| Participants with hip pain at baseline | | | | | | | | | | | |
| Events N (%) | 9  (1.9) | 5  (1.7) | 3  (1.4) | 0  (0.0) | 33  (2.1) | 34  (29.3) | 18  (26.9) | 11  (29.0) | 6  (26.1) | 60  (23.8) |  |
| Odds ratio  (95% CI) | 0.85  (0.38-1.89) | 0.62  (0.22-1.77) | 0.41  (0.10-1.74) | 0.13  (0.01-2.08) | 1  (reference) | 1.54  (0.88-2.68) | 1.49  (0.76-2.92) | 1.51  (0.67-3.39) | 1.28  (0.45-3.64) | 1  (reference) |  |
| **Osteophytes femoral inferior** | | | | | | | | | | | |
| All participants | | | | | | | | | | | |
| Events N (%) | 5  (0.6) | 2  (0.4) | 2  (0.5) | 2  (0.6) | 16  (0.4) | 37  (14.7) | 15  (10.7) | 10  (11.1) | 6  (9.7) | 93  (12.2) |  |
| Odds ratio  (95% CI) | 1.62  (0.57-4.58) | 0.88  (0.19-4.07) | 1.16  (0.25-5.34) | 1.46  (0.32-6.79) | 1  (reference) | 1.26  (0.80-1.97) | 0.88  (0.47-1.62) | 0.97  (0.46-2.02) | 0.82  (0.33-2.09) | 1  (reference) |  |
| Participants with hip pain at baseline | | | | | | | | | | | |
| Events N (%) | 2  (0.4) | 1  (0.3) | 1  (0.5) | 1  (0.6) | 6  (0.4) | 19  (16.4) | 7  (10.5) | 3  (7.9) | 1  (4.4) | 35  (13.9) |  |
| Odds ratio  (95% CI) | 1.17  (0.23-5.88) | 0.90  (0.11-7.56) | 1.19  (0.14-9.95) | 1.51  (0.18-12.70) | 1  (reference) | 1.22  (0.64-2.32) | 0.80  (0.33-1.93) | 0.64  (0.18-2.28) | 0.44  (0.05-3.50) | 1  (reference) |  |
| **PROGRESSION COHORTS** | | | | | | | | | | | |
| All participants | N=67 | N=32 | N=22 | N=18 | N=218 | N=40 | N=23 | N=12 | N=10 | N=66 |  |
| Participants with hip pain at baseline | N=37 | N=19 | N=14 | N=11 | N=103 | N=20 | N=13 | N=7 | N=5 | N=35 |  |
| **Joint space narrowing lateral** | | | | | | | | | | |  |
| All participants | | | | | | | | | | |  |
| Events N (%) | 7  (10.5) | 2  (6.3) | 2  (9.1) | 1  (5.6) | 26  (11.9) | 3  (7.5) | 1  (4.4) | 1  (8.3) | 1  (10.0) | 4  (6.1) |  |
| Odds ratio  (95% CI) | 0.97  (0.38-2.47) | 0.51  (0.10-2.45) | 0.89  (0.17-4.58) | 0.55  (0.06-5.06) | 1  (reference) | 2.01  (0.35-11.68) | 0.81  (0.04-18.36) | 7.18  (0.26-200.01) | 7.50  (0.27-208.11) | 1  (reference) |  |
| Participants with hip pain at baseline | | | | | | | | | | |  |
| Events N (%) | 3  (8.1) | 2  (10.5) | 2  (14.3) | 1  (9.1) | 14  (13.6) | 0  (0.0) | 0  (0.0) | 0  (0.0) | 0  (0.0) | 2  (5.7) |  |
| Odds ratio  (95% CI) | 0.65  (0.15-2.81) | 0.64  (0.11-3.68) | 1.17  (0.18-7.73 | 0.90  (0.08-9.80) | 1  (reference) | 0.93  (0.03-28.39) | 1.20  (0.02-73.63) | 2.44  (0.04-138.83) | 3.91  (0.05-280.85) | 1  (reference) |  |
| **Joint space narrowing medial** | | | | | | | | | | |  |
| All participants | | | | | | | | | | |  |
| Events N (%) | 9  (13.4) | 3  (9.4) | 2  (9.1) | 1  (5.6) | 32  (14.7) | 4  (10.0) | 2  (8.7) | 1  (8.3) | 0  (0.0) | 6  (9.1) |  |
| Odds ratio  (95% CI) | 1.01  (0.44-2.37) | 0.51  (0.10-2.45) | 0.67  (0.14-3.24) | 0.37  (0.05-3.08) | 1  (reference) | 2.74  (0.37-20.01) | 1.15  (0.06-21.17) | 17.85  (0.24-1346.97) | 1.99  (0.06-71.08) | 1  (reference) |  |
| Participants with hip pain at baseline | | | | | | | | | | |  |
| Events N (%) | 5  (13.5) | 3  (15.8) | 2  (14.3) | 1  (9.1) | 12  (11.7) | 0  (0.0) | 2  (15.4) | 1  (14.3) | 0  (0.0) | 5  (14.3) |  |
| Odds ratio  (95% CI) | 1.43  (0.42-4.84) | 0.64  (0.11-3.68) | 1.36  (0.24-7.78 | 0.85  (0.09-7.77) | 1  (reference) | 9.37  (0.51-170.81) | 3.13  (0.09-105.47) | 5.18  (0.26-103.14) | 2.32  (0.05-114.57) | 1  (reference) |  |
| **Total osteophytes score ≥ 2** | | | | | | | | | | |  |
| All participants | | | | | | | | | | |  |
| Events N (%) | 7  (10.5) | 2  (6.3) | 2  (9.1) | 1  (5.6) | 17  (7.8) | 10  (25.0) | 6  (26.1) | 3  (25.0) | 3  (30.0) | 10  (15.2) |  |
| Odds ratio  (95% CI) | 1.61  (0.61-4.28) | 0.85  (0.18-4.05) | 1.38  (0.28-6.86) | 0.80  (0.10-6.56) | 1  (reference) | 2.01  (0.63-6.38) | 1.75  (0.41-7.47) | 1.02  (0.17-6.10) | 2.09  (0.31-13.99) | 1  (reference) |  |
| Participants with hip pain at baseline | | | | | | | | | | |  |
| Events N (%) | 4  (10.8) | 2  (10.5) | 2  (14.3) | 1  (9.1) | 8  (7.8) | 3  (15.0) | 3  (23.1) | 1  (14.3) | 1  (20.0) | 8  (22.9) |  |
| Odds ratio  (95% CI) | 1.29  (0.32-5.16) | 1.04  (0.17-6.40) | 1.66  (0.25-11.16) | 0.99  (0.09-10.90) | 1  (reference) | 0.42  (0.05-3.31) | 0.57  (0.07-4.85) | 0.18  (0.01-3.07) | 0.41  (0.02-8.41) | 1  (reference) |  |
| **Osteophytes acetabular superior** | | | | | | | | | | |  |
| All participants | | | | | | | | | | |  |
| Events N (%) | 5  (7.5) | 1  (3.1) | 1  (4.6) | 0  (0.0) | 16  (7.3) | 12  (30.0) | 7  (30.4) | 3  (25.0) | 3  (30.0) | 12  (18.2) |  |
| Odds ratio  (95% CI) | 0.97  (0.30-3.10) | 0.44  (0.05-3.64) | 0.72  (0.09-6.04) | 0.34  (0.02-5.95) | 1  (reference) | 1.96  (0.65-5.88) | 2.49  (0.67-9.31) | 1.87  (0.36-9.68) | 3.20  (0.55-18.72) | 1  (reference) |  |
| Participants with hip pain at baseline | | | | | | | | | | |  |
| Events N (%) | 3  (8.1) | 1  (5.3) | 1  (7.1) | 0  (0.0) | 8  (7.8) | 4  (20.0) | 3  (23.1) | 1  (14.3) | 1  (20.0) | 7  (20.0) |  |
| Odds ratio  (95% CI) | 0.75  (0.14-4.06) | 0.66  (0.07-6.08) | 1.06  (0.11-10.50) | 0.53  (0.03-9.90) | 1  (reference) | 1.16  (0.21-6.55) | 1.28  (0.17-9.53) | 0.81  (0.06-10.83) | 1.75  (0.10-29.53) | 1  (reference) |  |
| **Osteophytes acetabular inferior** | | | | | | | | | | |  |
| All participants | | | | | | | | | | |  |
| Events N (%) | 2  (3.0) | 2  (6.3) | 2  (9.1) | 2  (11.1) | 11  (5.1) | 9  (22.5) | 5  (21.7) | 2  (16.7) | 2  (20.0) | 3  (4.6) |  |
| Odds ratio  (95% CI) | 0.49  (0.10-2.36) | 1.02  (0.20-5.22) | 1.62  (0.30-8.70) | 2.24  (0.42-11.87) | 1  (reference) | 11.01  (2.04-59.53) | 8.30  (1.24-55.81) | 3.98  (0.47-34.03) | 6.31  (0.69-57.58) | 1  (reference) |  |
| Participants with hip pain at baseline | | | | | | | | | | |  |
| Events N (%) | 1  (2.7) | 1  (5.3) | 1  (7.1) | 1  (9.1) | 6  (5.8) | 5  (25.0) | 3  (23.1) | 0  (0.0) | 0  (0.0) | 3  (8.6) |  |
| Odds ratio  (95% CI) | 0.36  (0.04-3.49) | 0.70  (0.07-7.35) | 1.08  (0.10-12.32) | 1.48  (0.13-16.52) | 1  (reference) | 3.54  (0.50-24.89) | 2.24  (0.22-22.85) | 0.35  (0.01-8.20) | 0.52  (0.02-13.01) | 1  (reference) |  |
| **Osteophytes femoral superior** | | | | | | | | | | |  |
| All participants | | | | | | | | | | |  |
| Events N (%) | 8  (11.9) | 3  (9.4) | 2  (9.1) | 1  (5.6) | 21  (9.6) | 14  (35.0) | 8  (34.8) | 5  (41.7) | 4  (40.0) | 15  (22.7) |  |
| Odds ratio  (95% CI) | 1.46  (0.56-3.80) | 0.96  (0.23-4.02) | 1.08  (0.21-5.66) | 0.59  (0.07-5.17) | 1  (reference) | 2.16  (0.84-5.52) | 1.99  (0.62-6.41) | 3.56  (0.85-14.86) | 4.10  (0.86-19.58) | 1  (reference) |  |
| Participants with hip pain at baseline | | | | | | | | | | |  |
| Events N (%) | 4  (10.8) | 2  (10.5) | 1  (7.1) | 0  (0.0) | 11  (10.7) | 8  (40.0) | 6  (46.2) | 3  (42.9) | 2  (40.0) | 8  (22.9) |  |
| Odds ratio  (95% CI) | 0.97  (0.23-4.00) | 0.68  (0.09-5.06) | 0.67  (0.06-6.93) | 0.33  (0.02-7.15) | 1  (reference) | 3.46  (0.65-18.39) | 4.14  (0.61-27.89) | 4.03  (0.44-36.90) | 6.72  (0.35-128.56) | 1  (reference) |  |
| **Osteophytes femoral inferior** | | | | | | | | | | |  |
| All participants | | | | | | | | | | |  |
| Events N (%) | 2  (3.0) | 1  (3.1) | 1  (4.6) | 1  (2.0) | 22  (10.1) | 8  (20.0) | 7  (30.4) | 3  (25.0) | 3  (30.0) | 9  (13.6) |  |
| Odds ratio  (95% CI) | 0.33  (0.07-1.45) | 0.33  (0.04-2.65) | 0.53  (0.06-4.38) | 0.61  (0.07-5.10) | 1  (reference) | 1.43  (0.45-4.53) | 2.38  (0.65-8.68) | 1.48  (0.31-6.99) | 2.01  (0.40-10.05) | 1  (reference) |  |
| Participants with hip pain at baseline | | | | | | | | | | |  |
| Events N (%) | 2  (5.4) | 1  (5.3) | 1  (7.1) | 1  (9.1) | 7  (6.8) | 5  (25.0) | 5  (38.5) | 2  (28.6) | 2  (40.0) | 4  (11.4) |  |
| Odds ratio  (95% CI) | 0.80  (0.14-4.57) | 0.70  (0.07-7.23) | 1.03  (0.09-11.38) | 1.29  (0.12-14.04) | 1  (reference) | 1.03  (0.15-6.96) | 1.85  (0.25-13.99) | 1.11  (0.10-12.56) | 3.25  (0.18-60.01) | 1  (reference) |  |

The analysis was adjusted for: age at baseline; sex; body mass index at baseline, and comorbidity score at baseline. CHECK: Cohort of Hip and Cohort of Knee; CI: Confidence interval; NSAIDs: Non-Steroidal Anti-Inflammatory Drugs; OA: Osteoarthritis; OAI: Osteoarthritis Initiative.

**Table S2.** Association of long-term use of NSAIDs by categorizing NSAID use into cumulative timeframes and degeneration of individual radiographic features of the hip, during 4 to 5 years follow-up (sensitivity analysis)

| Outcome | OAI | | | | | CHECK | | | | |
| --- | --- | --- | --- | --- | --- | --- | --- | --- | --- | --- |
|  | NSAID users  ≥ 1-year | NSAID users  ≥ 2-year | NSAID users  ≥ 3-year | NSAID users  ≥ 4-year | NSAID  non-users | NSAID users  ≥ 1-year | NSAID users  ≥ 2-year | NSAID users  ≥ 3-year | NSAID users  ≥ 4-year | NSAID  non-users |
| **INCIDENCE COHORTS** | | | | | | | | | | |
| All participants | N=892 | N=576 | N=415 | N=314 | N=4,261 | N=251 | N=140 | N=90 | N=62 | N=760 |
| Participants with hip pain at baseline | N=464 | N=303 | N=219 | N=167 | N=1,543 | N=116 | N=67 | N=38 | N=23 | N=252 |
| **Joint space narrowing lateral** | | | | | | | | | | |
| All participants | | | | | | | | | | |
| Events N (%) | 9  (1.0) | 7  (1.2) | 4  (1.0) | 3  (1.0) | 72  (1.7) | 17  (6.8) | 11  (7.9) | 5  (5.6) | 3  (4.8) | 66  (8.7) |
| Odds ratio  (95% CI) | 0.64  (0.31-1.31) | 0.77  (0.35-1.72) | 0.62  (0.22-1.72) | 0.61  (0.19-1.98) | 1  (reference) | 0.90  (0.49-1.64) | 1.12  (0.54-2.34) | 0.77  (0.28-2.11) | 0.66  (0.19-2.36) | 1  (reference) |
| Participants with hip pain at baseline | | | | | | | | | | |
| Events N (%) | 6  (1.3) | 5  (1.7) | 4  (1.8) | 3  (1.8) | 29  (1.9) | 17  (14.7) | 3  (4.5) | 0  (0.0) | 0  (0.0) | 21  (8.3) |
| Odds ratio  (95% CI) | 0.94  (0.38-2.33) | 1.21  (0.46-3.22) | 1.37  (0.47-4.01) | 1.36  (0.40-4.59) | 1  (reference) | 0.92  (0.38-2.24) | 0.67  (0.18-2.45) | 0.17  (0.01-2.91) | 0.28  (0.02-4.98) | 1  (reference) |
| **Joint space narrowing medial** | | | | | | | | | | |
| All participants | | | | | | | | | | |
| Events N (%) | 35  (3.9) | 19  (3.3) | 18  (4.3) | 14  (4.5) | 119  (2.8) | 48  (19.1) | 25  (17.9) | 20  (22.2) | 11  (17.7) | 134  (17.6) |
| Odds ratio  (95% CI) | 1.47  (0.96-2.25) | 1.16  (0.67-2.00) | 1.50  (0.86-2.64) | 1.89  (0.80-2.80) | 1  (reference) | 1.19  (0.77-1.84) | 1.06  (0.61-1.85) | 1.39  (0.75-2.59) | 1.06  (0.48-2.34) | 1  (reference) |
| Participants with hip pain at baseline | | | | | | | | | | |
| Events N (%) | 20  (4.3) | 13  (4.3) | 12  (5.5) | 9  (5.4) | 46  (3.0) | 25  (21.6) | 15  (22.4) | 11  (29.0) | 5  (21.7) | 43  (17.1) |
| Odds ratio  (95% CI) | 1.70  (0.95-3.03) | 1.68  (0.85-3.13) | 2.09  (1.02-4.25) | 1.89  (0.84-4.24) | 1  (reference) | 1.49  (0.81-2.75) | 1.55  (0.76-3.17) | **2.32**  **(1.02-5.28)** | 1.76  (0.59-5.23) | 1  (reference) |
| **Total osteophytes score ≥ 2** | | | | | | | | | | |
| All participants | | | | | | | | | | |
| Events N (%) | 8  (0.9) | 2  (0.4) | 1  (0.2) | 1  (0.3) | 26  (0.6) | 38  (15.1) | 20  (14.3) | 14  (15.6) | 10  (16.1) | 136  (17.9) |
| Odds ratio  (95% CI) | 1.55  (0.66-3.65) | 0.57  (0.13-2.54) | 0.36  (0.04-2.98) | 0.45  (0.05-3.78) | 1  (reference) | 0.90  (0.80-1.97) | 0.90  (0.51-1.61) | 1.04  (0.53-2.04) | 0.99  (0.44-2.22) | 1  (reference) |
| Participants with hip pain at baseline | | | | | | | | | | |
| Events N (%) | 5  (1.1) | 1  (0.3) | 0  (0.0) | 0  (0.0) | 13  (0.8) | 20  (17.2) | 9  (13.4) | 5  (13.2) | 2  (8.7) | 49  (19.4) |
| Odds ratio  (95% CI) | 1.47  (0.50-4.32) | 0.43  (0.06-3.29) | 0.27  (0.16-4.53) | 0.33  (0.02-5.62) | 1  (reference) | 1.06  (0.64-2.32) | 1.00  (0.44-2.31) | 1.13  (0.39-3.27) | 0.73  (0.16-3.46) | 1  (reference) |
| **Osteophytes acetabular superior** | | | | | | | | | | |
| All participants | | | | | | | | | | |
| Events N (%) | 16  (1.8) | 12  (2.1) | 10  (2.4) | 7  (2.2) | 56  (1.3) | 53  (21.1) | 25  (17.9) | 18  (20.0) | 15  (24.2) | 217  (28.6) |
| Odds ratio  (95% CI) | 1.38  (0.75-2.55) | 1.63  (0.83-3.20) | 1.81  (0.87-3.78) | 1.69  (0.72-3.94) | 1  (reference) | 0.69  (0.47-1.03) | 0.56  (0.33-0.95) | 0.65  (0.35-1.21) | 0.83  (0.42-1.65) | 1  (reference) |
| Participants with hip pain at baseline | | | | | | | | | | |
| Events N (%) | 10  (2.2) | 6  (2.0) | 4  (1.8) | 2  (1.2) | 28  (1.8) | 26  (22.4) | 14  (20.9) | 10  (26.3) | 7  (30.4) | 77  (30.6) |
| Odds ratio  (95% CI) | 1.29  (0.59-2.80) | 1.22  (0.48-3.09) | 1.01  (0.32-3.26) | 0.67  (0.16-2.89) | 1  (reference) | 0.72  (0.41-1.27) | 0.71  (0.35-1.42) | 0.91  (0.40-2.09) | 1.29  (0.48-3.50) | 1  (reference) |
| **Osteophytes acetabular inferior** | | | | | | | | | | |
| All participants | | | | | | | | | | |
| Events N (%) | 3  (0.3) | 0  (0.0) | 0  (0.0) | 0  (0.0) | 19  (0.5) | 15  (6.0) | 9  (6.4) | 5  (5.6) | 3  (4.8) | 65  (8.6) |
| Odds ratio  (95% CI) | 0.76  (0.21-2.84) | 0.17  (0.01-2.89) | 0.24  (0.01-3.94) | 0.30  (0.02-5.80) | 1  (reference) | 0.78  (0.41-1.48) | 0.89  (0.41-1.95) | 0.76  (0.28-2.09) | 0.64  (0.18-2.31) | 1  (reference) |
| Participants with hip pain at baseline | | | | | | | | | | |
| Events N (%) | 0  (0.0) | 0  (0.0) | 0  (0.0) | 0  (0.0) | 9  (0.6) | 9  (7.8) | 5  (7.5) | 2  (5.3) | 1  (4.4) | 20  (7.9) |
| Odds ratio  (95% CI) | 0.16  (0.01-2.79) | 0.25  (0.01-4.38) | 0.36  (0.02-6.17) | 0.47  (0.03-8.07) | 1  (reference) | 1.16  (0.47-2.86) | 1.50  (0.50-4.50) | 1.04  (0.22-4.92) | 0.89  (0.11-7.42) | 1  (reference) |
| **Osteophytes femoral superior** | | | | | | | | | | |
| All participants | | | | | | | | | | |
| Events N (%) | 18  (2.0) | 12  (2.1) | 8  (1.9) | 4  (1.3) | 72  (1.7) | 68  (27.1) | 37  (26.4) | 25  (27.8) | 18  (29.0) | 184  (24.2) |
| Odds ratio  (95% CI) | 1.09  (0.62-1.92) | 1.03  (0.52-2.04) | 0.83  (0.35-1.95) | 0.72  (0.26-2.01) | 1  (reference) | 1.25  (0.86-1.82) | 1.26  (0.79-2.01) | 1.31  (0.76-2.27) | 1.39  (0.73-2.63) | 1  (reference) |
| Participants with hip pain at baseline | | | | | | | | | | |
| Events N (%) | 9  (1.9) | 5  (1.7) | 3  (1.4) | 0  (0.0) | 33  (2.1) | 34  (29.3) | 18  (26.9) | 11  (29.0) | 6  (26.1) | 60  (23.8) |
| Odds ratio  (95% CI) | 0.85  (0.38-1.89) | 0.62  (0.22-1.77) | 0.41  (0.10-1.74) | 0.13  (0.01-2.08) | 1  (reference) | 1.54  (0.88-2.68) | 1.49  (0.76-2.92) | 1.51  (0.67-3.39) | 1.28  (0.45-3.64) | 1  (reference) |
| **Osteophytes femoral inferior** | | | | | | | | | | |
| All participants | | | | | | | | | | |
| Events N (%) | 5  (0.6) | 2  (0.4) | 2  (0.5) | 2  (0.6) | 16  (0.4) | 37  (14.7) | 15  (10.7) | 10  (11.1) | 6  (9.7) | 93  (12.2) |
| Odds ratio  (95% CI) | 1.62  (0.57-4.58) | 0.88  (0.19-4.07) | 1.16  (0.25-5.34) | 1.46  (0.32-6.79) | 1  (reference) | 1.26  (0.80-1.97) | 0.88  (0.47-1.62) | 0.97  (0.46-2.02) | 0.82  (0.33-2.09) | 1  (reference) |
| Participants with hip pain at baseline | | | | | | | | | | |
| Events N (%) | 2  (0.4) | 1  (0.3) | 1  (0.5) | 1  (0.6) | 6  (0.4) | 19  (16.4) | 7  (10.5) | 3  (7.9) | 1  (4.4) | 35  (13.9) |
| Odds ratio  (95% CI) | 1.17  (0.23-5.88) | 0.90  (0.11-7.56) | 1.19  (0.14-9.95) | 1.51  (0.18-12.70) | 1  (reference) | 1.22  (0.64-2.32) | 0.80  (0.33-1.93) | 0.64  (0.18-2.28) | 0.44  (0.05-3.50) | 1  (reference) |
| **PROGRESSION COHORTS** | | | | | | | | | | |
| All participants | N=67 | N=32 | N=22 | N=18 | N=218 | N=40 | N=23 | N=12 | N=10 | N=66 |
| Participants with hip pain at baseline | N=37 | N=19 | N=14 | N=11 | N=103 | N=20 | N=13 | N=7 | N=5 | N=35 |
| **Joint space narrowing lateral** | | | | | | | | | | |
| All participants | | | | | | | | | | |
| Events N (%) | 7  (10.5) | 2  (6.3) | 2  (9.1) | 1  (5.6) | 26  (11.9) | 3  (7.5) | 1  (4.4) | 1  (8.3) | 1  (10.0) | 4  (6.1) |
| Odds ratio  (95% CI) | 0.97  (0.38-2.47) | 0.51  (0.10-2.45) | 0.89  (0.17-4.58) | 0.55  (0.06-5.06) | 1  (reference) | 2.01  (0.35-11.68) | 0.81  (0.04-18.36) | 7.18  (0.26-200.01) | 7.50  (0.27-208.11) | 1  (reference) |
| Participants with hip pain at baseline | | | | | | | | | | |
| Events N (%) | 3  (8.1) | 2  (10.5) | 2  (14.3) | 1  (9.1) | 14  (13.6) | 0  (0.0) | 0  (0.0) | 0  (0.0) | 0  (0.0) | 2  (5.7) |
| Odds ratio  (95% CI) | 0.65  (0.15-2.81) | 0.64  (0.11-3.68) | 1.17  (0.18-7.73 | 0.90  (0.08-9.80) | 1  (reference) | 0.93  (0.03-28.39) | 1.20  (0.02-73.63) | 2.44  (0.04-138.83) | 3.91  (0.05-280.85) | 1  (reference) |
| **Joint space narrowing medial** | | | | | | | | | | |
| All participants | | | | | | | | | | |
| Events N (%) | 9  (13.4) | 3  (9.4) | 2  (9.1) | 1  (5.6) | 32  (14.7) | 4  (10.0) | 2  (8.7) | 1  (8.3) | 0  (0.0) | 6  (9.1) |
| Odds ratio  (95% CI) | 1.01  (0.44-2.37) | 0.51  (0.10-2.45) | 0.67  (0.14-3.24) | 0.37  (0.05-3.08) | 1  (reference) | 2.74  (0.37-20.01) | 1.15  (0.06-21.17) | 17.85  (0.24-1346.97) | 1.99  (0.06-71.08) | 1  (reference) |
| Participants with hip pain at baseline | | | | | | | | | | |
| Events N (%) | 5  (13.5) | 3  (15.8) | 2  (14.3) | 1  (9.1) | 12  (11.7) | 0  (0.0) | 2  (15.4) | 1  (14.3) | 0  (0.0) | 5  (14.3) |
| Odds ratio  (95% CI) | 1.43  (0.42-4.84) | 0.64  (0.11-3.68) | 1.36  (0.24-7.78 | 0.85  (0.09-7.77) | 1  (reference) | 9.37  (0.51-170.81) | 3.13  (0.09-105.47) | 5.18  (0.26-103.14) | 2.32  (0.05-114.57) | 1  (reference) |
| **Total osteophytes score ≥ 2** | | | | | | | | | | |
| All participants | | | | | | | | | | |
| Events N (%) | 7  (10.5) | 2  (6.3) | 2  (9.1) | 1  (5.6) | 17  (7.8) | 10  (25.0) | 6  (26.1) | 3  (25.0) | 3  (30.0) | 10  (15.2) |
| Odds ratio  (95% CI) | 1.61  (0.61-4.28) | 0.85  (0.18-4.05) | 1.38  (0.28-6.86) | 0.80  (0.10-6.56) | 1  (reference) | 2.01  (0.63-6.38) | 1.75  (0.41-7.47) | 1.02  (0.17-6.10) | 2.09  (0.31-13.99) | 1  (reference) |
| Participants with hip pain at baseline | | | | | | | | | | |
| Events N (%) | 4  (10.8) | 2  (10.5) | 2  (14.3) | 1  (9.1) | 8  (7.8) | 3  (15.0) | 3  (23.1) | 1  (14.3) | 1  (20.0) | 8  (22.9) |
| Odds ratio  (95% CI) | 1.29  (0.32-5.16) | 1.04  (0.17-6.40) | 1.66  (0.25-11.16) | 0.99  (0.09-10.90) | 1  (reference) | 0.42  (0.05-3.31) | 0.57  (0.07-4.85) | 0.18  (0.01-3.07) | 0.41  (0.02-8.41) | 1  (reference) |
| **Osteophytes acetabular superior** | | | | | | | | | | |
| All participants | | | | | | | | | | |
| Events N (%) | 5  (7.5) | 1  (3.1) | 1  (4.6) | 0  (0.0) | 16  (7.3) | 12  (30.0) | 7  (30.4) | 3  (25.0) | 3  (30.0) | 12  (18.2) |
| Odds ratio  (95% CI) | 0.97  (0.30-3.10) | 0.44  (0.05-3.64) | 0.72  (0.09-6.04) | 0.34  (0.02-5.95) | 1  (reference) | 1.96  (0.65-5.88) | 2.49  (0.67-9.31) | 1.87  (0.36-9.68) | 3.20  (0.55-18.72) | 1  (reference) |
| Participants with hip pain at baseline | | | | | | | | | | |
| Events N (%) | 3  (8.1) | 1  (5.3) | 1  (7.1) | 0  (0.0) | 8  (7.8) | 4  (20.0) | 3  (23.1) | 1  (14.3) | 1  (20.0) | 7  (20.0) |
| Odds ratio  (95% CI) | 0.75  (0.14-4.06) | 0.66  (0.07-6.08) | 1.06  (0.11-10.50) | 0.53  (0.03-9.90) | 1  (reference) | 1.16  (0.21-6.55) | 1.28  (0.17-9.53) | 0.81  (0.06-10.83) | 1.75  (0.10-29.53) | 1  (reference) |
| **Osteophytes acetabular inferior** | | | | | | | | | | |
| All participants | | | | | | | | | | |
| Events N (%) | 2  (3.0) | 2  (6.3) | 2  (9.1) | 2  (11.1) | 11  (5.1) | 9  (22.5) | 5  (21.7) | 2  (16.7) | 2  (20.0) | 3  (4.6) |
| Odds ratio  (95% CI) | 0.49  (0.10-2.36) | 1.02  (0.20-5.22) | 1.62  (0.30-8.70) | 2.24  (0.42-11.87) | 1  (reference) | 11.01  (2.04-59.53) | 8.30  (1.24-55.81) | 3.98  (0.47-34.03) | 6.31  (0.69-57.58) | 1  (reference) |
| Participants with hip pain at baseline | | | | | | | | | | |
| Events N (%) | 1  (2.7) | 1  (5.3) | 1  (7.1) | 1  (9.1) | 6  (5.8) | 5  (25.0) | 3  (23.1) | 0  (0.0) | 0  (0.0) | 3  (8.6) |
| Odds ratio  (95% CI) | 0.36  (0.04-3.49) | 0.70  (0.07-7.35) | 1.08  (0.10-12.32) | 1.48  (0.13-16.52) | 1  (reference) | 3.54  (0.50-24.89) | 2.24  (0.22-22.85) | 0.35  (0.01-8.20) | 0.52  (0.02-13.01) | 1  (reference) |
| **Osteophytes femoral superior** | | | | | | | | | | |
| All participants | | | | | | | | | | |
| Events N (%) | 8  (11.9) | 3  (9.4) | 2  (9.1) | 1  (5.6) | 21  (9.6) | 14  (35.0) | 8  (34.8) | 5  (41.7) | 4  (40.0) | 15  (22.7) |
| Odds ratio  (95% CI) | 1.46  (0.56-3.80) | 0.96  (0.23-4.02) | 1.08  (0.21-5.66) | 0.59  (0.07-5.17) | 1  (reference) | 2.16  (0.84-5.52) | 1.99  (0.62-6.41) | 3.56  (0.85-14.86) | 4.10  (0.86-19.58) | 1  (reference) |
| Participants with hip pain at baseline | | | | | | | | | | |
| Events N (%) | 4  (10.8) | 2  (10.5) | 1  (7.1) | 0  (0.0) | 11  (10.7) | 8  (40.0) | 6  (46.2) | 3  (42.9) | 2  (40.0) | 8  (22.9) |
| Odds ratio  (95% CI) | 0.97  (0.23-4.00) | 0.68  (0.09-5.06) | 0.67  (0.06-6.93) | 0.33  (0.02-7.15) | 1  (reference) | 3.46  (0.65-18.39) | 4.14  (0.61-27.89) | 4.03  (0.44-36.90) | 6.72  (0.35-128.56) | 1  (reference) |
| **Osteophytes femoral inferior** | | | | | | | | | | |
| All participants | | | | | | | | | | |
| Events N (%) | 2  (3.0) | 1  (3.1) | 1  (4.6) | 1  (2.0) | 22  (10.1) | 8  (20.0) | 7  (30.4) | 3  (25.0) | 3  (30.0) | 9  (13.6) |
| Odds ratio  (95% CI) | 0.33  (0.07-1.45) | 0.33  (0.04-2.65) | 0.53  (0.06-4.38) | 0.61  (0.07-5.10) | 1  (reference) | 1.43  (0.45-4.53) | 2.38  (0.65-8.68) | 1.48  (0.31-6.99) | 2.01  (0.40-10.05) | 1  (reference) |
| Participants with hip pain at baseline | | | | | | | | | | |
| Events N (%) | 2  (5.4) | 1  (5.3) | 1  (7.1) | 1  (9.1) | 7  (6.8) | 5  (25.0) | 5  (38.5) | 2  (28.6) | 2  (40.0) | 4  (11.4) |
| Odds ratio  (95% CI) | 0.80  (0.14-4.57) | 0.70  (0.07-7.23) | 1.03  (0.09-11.38) | 1.29  (0.12-14.04) | 1  (reference) | 1.03  (0.15-6.96) | 1.85  (0.25-13.99) | 1.11  (0.10-12.56) | 3.25  (0.18-60.01) | 1  (reference) |

The analysis was adjusted for: age at baseline; sex; body mass index at baseline, and comorbidity score at baseline. CHECK: Cohort of Hip and Cohort of Knee; CI: Confidence interval; NSAIDs: Non-Steroidal Anti-Inflammatory Drugs; OA: Osteoarthritis; OAI: Osteoarthritis Initiative.

END OF DOCUMENT
